# Validity and Reliability of Three Commercially Available Smart Sports Bras during Treadmill Walking and Running

**DOI:** 10.1038/s41598-020-64185-z

**Published:** 2020-04-30

**Authors:** James W. Navalta, Gabriela Guzman Ramirez, Crystal Maxwell, Kara N. Radzak, Graham R. McGinnis

**Affiliations:** University of Nevada, Las Vegas, Department of Kinesiology and Nutrition Sciences, Las Vegas, Nevada 89154 USA

**Keywords:** Cardiovascular biology, Cardiovascular biology

## Abstract

A variety of wearable technology devices report heart rate. Heart rate sensing smart bras are manufactured for females who participate in activity, however accuracy has not been determined. The purpose was to determine the validity of heart rate measures in three commercially available sports bras during walking and running. Twenty-four healthy females completed bouts of treadmill exercise. The Adidas Smart sports bra, Berlei sports bra, and Sensoria Fitness biometric sports bra were tested. Participant perception of each garment was obtained immediately after the participant divested the sports bra. The Adidas Smart sports bra was valid only during rest (Intraclass correlation Coefficient [ICC] = 0.79, mean absolute percentage error [MAPE] = 4.5%, Limits of Agreement [LoA]=−8 to 8). The Berlei sports bra was valid across all conditions (ICC = 0.99, MAPE = 0.66%, LoA = −19 to 19), and the Sensoria biometric bra was valid during rest and walking (ICC = 0.96, MAPE = 1.9%, LoA = −15 to 12). Perception of the smart sports bras was higher for the Adidas Smart sports bra and Sensoria Fitness sports bra, and lower for the Berlei sports bra. Sports bra manufacturers designing wearable technology garments must consider the ability of returning accurate biometric data while providing necessary function and comfort to females engaging in physical activity.

## Introduction

The use of commercially available wearable technology devices has increased worldwide over the past few years as indicated by the demand for such products (unit shipments 2015 = 81.9 million, 2016 = 102.4 million, 2017 = 115.4 million, 2018 = 172.2 million)^1^. As such, the volume of research dedicated to wearable technology has increased at a similar pace (2015 = 671 manuscripts, 2016 = 975 manuscripts, 2017 = 1575 manuscripts, 2018 = 1575 manuscripts). These wearable devices return a variety of measures including heart rate, step count, energy expenditure, and many more. However, there often appears to be a disagreement with the criterion measurements of these variables, revealing a hierarchy with respect to accuracy. In support, we have previously shown that heart rate returns the most valid measures, energy expenditure has the least valid measures, and step count falls somewhere in between^2^.

A number of wearable technology devices with the ability to return heart rate have been investigated, including smart shirts^3–7^, earbuds^8–10^ and sensors placed around the forearm^11–13^ or wrist^14–18^. While smart shirts are valid and reliable during laboratory-based walking (validity Intraclass Correlation Coefficient [ICC] range = 0.81 to 0.99, reliability range = 0.85 to 0.88)^4,7^ and cycling (validity range = 0.98 to 0.99, reliability range = 0.94 to 0.96)^5^, reliability (range = 0.65 to 0.73)^3^ and accuracy decreased when utilized in an outdoor environment (range = −0.012 to 0.354)^6^. Heart rate sensing earbuds have relatively little literature, however the general trend is that validity is high with low intensity cycling (ICC = 0.97)^8^, treadmill exercise (ICC = 0.98)^10^ or running (ICC = 0.953)^9^, but that accuracy decreases with greater intensity (100% cycle intensity ICC = 0.50)^8^, (high intensity training ICC = 0.861)^9^. Similarly, forearm-located sensors are accurate during treadmill and cycle exercise (ICC range 0.84 to 0.93)^11–13^, but decrease as the action becomes more vigorous (ICC range 0.27 to 0.41)^12^. The largest volume of literature can be found on wrist-worn devices, and incorporates a variety of exercise protocols^2,8,12,14–19^. Despite the wide array of wrist-worn devices utilized, validity has been shown to be good with ICC ranges between 0.74^14^ and 0.947^16^. The primary limitation with heart rate returned from wrist-worn devices is motion artifact in the photoplethysmography wave form that decreases accuracy as exercise intensity increases^20^. Other limitations with this measurement technique include effects of skin tone^21^, the influence of ambient light^22^, and no universally accepted standards for clinical measurements^23^. Heart rate strap monitors may be considered one of the earliest “wearable” technology devices used during activity. These type of monitors have been validated against pulse oximeters and electrocardiograms in returning accurate heart rate values^8,12,24–26^ and are also reliable during exercise^27^. The ability to place sensors in a garment already being worn for physical activity, such as the sports bra for women, makes it an attractive target for biometric data collection.

To date, no heart rate sensing sports bra has been validated in the wearable technology literature. The aim of utilizing a sports bra during exercise is to provide appropriate breast support to reduce exercise associated breast pain^28^, while maintaining a comfortable fit. A properly fitted bra is important, as breast development and size may present a barrier to physical activity and exercise from an early age^29^ and into adulthood^30^. Unfortunately, women rarely wear the correct bra size and the vast majority (85%) are unable to independently choose a well-fitted bra^31^. When considering exercise, ratings of exercise-induced pain and discomfort are reported to be lower when wearing highly supportive, compared to wearing low supporting, garments^32^. There is a need for designers of sports bras to consider how these garments can reduce force generation and discomfort by providing high levels of support during activity^33^. Thus, as wearable technology sensors are introduced into sports bras, there is a need to determine not only the technical capabilities of the sensor, but also the functional aspects of the garment.

The concept of a heart rate sensing brassiere was reported in 2014^34^, and efforts have been made to utilize technology-sensing bras to detect breast cancer^35,36^. However, to our knowledge there have been no published investigations that have assessed the validity or reliability of heart rate sensing bras during exercise. Thus, the primary purpose of this investigation was to determine heart rate validity and reliability measurements in three commercially available sports bras. It was hypothesized that three different brands of smart bra would be both valid and reliable. A secondary purpose was to obtain subjective measures regarding perceptions of these sports bras from participants.

## Results

Validity measures are reported in Table 1, and partitioned into the activity types of standing rest, self-paced warm up, self-paced run, and self-paced walk. Additionally, validation throughout the entire period is presented (noted as “All’). Self-paced speeds utilized by participants are presented in Table 2, and no differences were noted in pace between sports bra trials. The percent of erroneous heart rate values produced by each sports bra for each condition is displayed in Table 3.

**Table 1:** Second-by-second validation of heart rate obtained from three smart bras compared to a criterion measure over various conditions (rest, warm up, run, walk, and over the entire period). Validation tests include mean absolute percentage error (MAPE), Bland-Altman limits of agreement with (associated 95% confidence intervals), and Intraclass correlations (ICC) with associated 95% confidence intervals (CI) and p-values.

Smart Bra	Condition	MAPE (%)	Limits of Agreement (95% CI)	ICC	95% CI	p-value
Adidas	**Rest**	**4.45**	**−8 (−19, 2) to 8 (−2, 19)**	**0.792**	**0.769 to 0.813**	**<0.001**
	Warm Up	9.32	54 (−64, −43) to 61 (50, 71)	0.600	0.575 to 0.623	<0.001
	Run	13.57	−47 (−63, −30) to 57 (40, 74)	0.531	0.509 to 0.553	<0.001
	Walk	9.56	−52 (−74, −30) to 71 (49, 93)	0.572	0.552 to 0.592	<0.001
	All	10.56	−192 (−203, −181) to 208 (197, 220)	0.662	0.652 to 0.671	<0.001
Berlei	**Rest**	**0.93**	**−2 (−12, 8) to 2 (−8, 12)**	**0.997**	**0.996 to 0.997**	**<0.001**
	**Warm Up**	**0.78**	**−5 (−10, 1) to 5 (−1, 10)**	**0.997**	**0.997 to 0.997**	**<0.001**
	**Run**	**0.58**	**−5 (−10, 1) to 5 (−1, 11)**	**0.996**	**0.995 to 0.996**	**<0.001**
	**Walk**	**0.61**	**−2 (−14, 10) to 2 (−10, 14)**	**0.999**	**0.999 to 0.999**	**<0.001**
	**All**	**0.66**	**−19 (−20, −17) to 19 (18, 20)**	**0.998**	**0.998 to 0.998**	**<0.001**
Sensoria	**Rest**	**2.26**	**−10 (−12, −9) to 8 (7, 10)**	**0.969**	**0.965 to 0.972**	**<0.001**
	Warm Up	5.32	−45 (−55, −36) to 48 (39, 58)	0.696	0.677 to 0.715	<0.001
	Run	4.00	−47 (−61, −33) to 58 (44, 73)	0.628	0.610 to 0.646	<0.001
	**Walk**	**1.91**	**−15 (−17, −13) to 12 (10, 14)**	**0.958**	**0.956 to 0.960**	**<0.001**
	All	3.40	−151 (−156, −146) to 154 (149, 159)	0.840	0.836 to 0.845	<0.001

Note: bold indicates having met the minimum established thresholds for each of the validity tests.

**Table 2:** Self-paced speed (expressed in meters per minute) employed by participants during the warm up, run, and walk portions of the protocol. Data are presented as average ± standard error.

	Warm up	Run	Walk
Adidas	83.15 ± 8.39	139.59 ± 6.85	63.21 ± 2.47
Berlei	90.15 ± 9.30	142.39 ± 8.12	66.82 ± 2.57
Sensoria	90.61 ± 9.89	142.98 ± 8.68	66.36 ± 2.56
p-value	0.881	0.959	0.658

**Table 3:** Percent of erroneous heart rate values produced during each condition.

	Rest	Warm up	Run	Walk
Adidas	0.004%	0.08%	0.04%	0.09%
Berlei	0.0%	0.0%	0.007%	0.0%
Sensoria	0.008%	0.03%	0.04%	0.0005%

**Figure 1:**
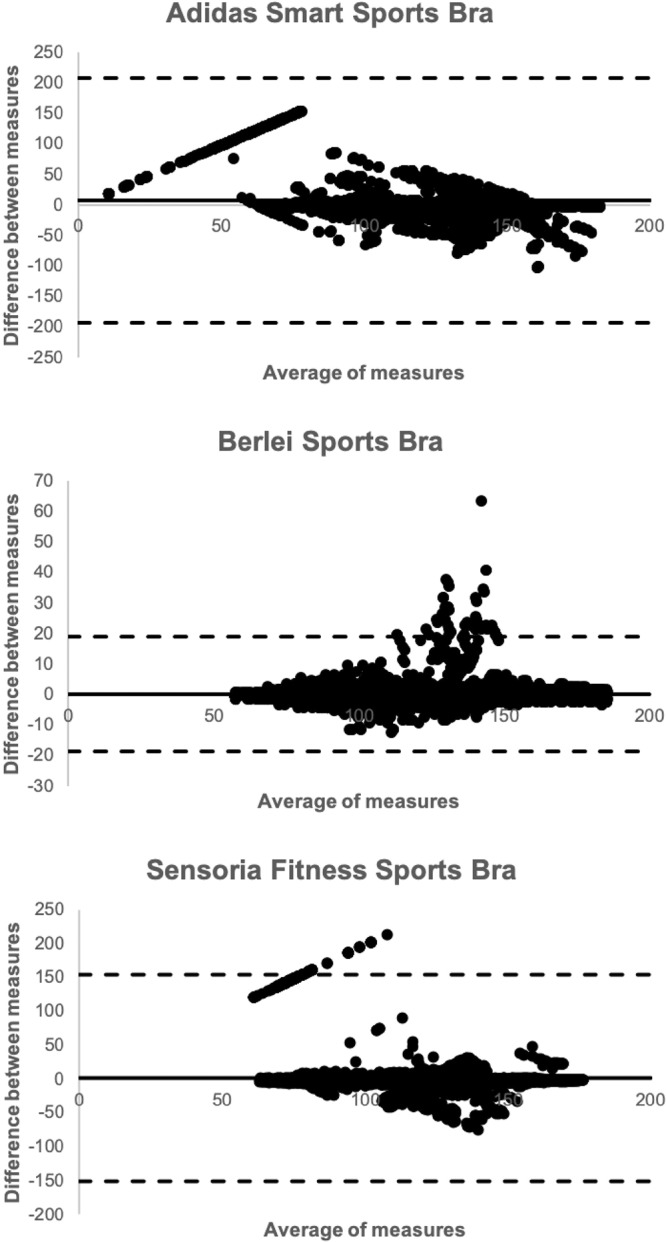
Bland-Altman plots associated with the Adidas Smart sports bra, Berlei sports bra, and Sensoria Fitness biometric sports bra.

The Adidas Smart sports bra was considered valid (Mean Absolute Percent Error <5%, Intraclass Correlation Coefficient > 0.70) only for the resting condition. The Berlei sports bra met the minimum criterion thresholds for all validity tests for every condition. The Sensoria Fitness biometric sports bra was considered valid during rest and during the self-paced walk. Bland-Altman plots are shown in Fig. 1.

Reliability measures of each smart bra for the different conditions in returning consistent heart rates are reported in Table 4. The Adidas Smart sports bra was not considered to be reliable for any condition. The Berlei sports bra met the minimum criterion thresholds for all reliability tests for every condition. The Sensoria Fitness biometric sports bra was considered reliable in the resting condition.

**Table 4:** Second-by-second reliability of heart rate obtained from three smart bras over various conditions (rest, warm up, run, walk, and over the entire period). Absolute reliability is represented as the coefficient of variation (CV), and test-retest reliability is presented as Intraclass correlations (ICC) with associated 95% confidence intervals (CI) and p-values.

Smart Bra	Condition	CV (%)	ICC	95% CI	p-value
Adidas	Rest	10.23	0.551	0.501 to 0.595	<0.001
	Warm Up	19.66	0.337	0.295 to 0.376	<0.001
	Run	24.12	0.155	0.114 to 0.194	<0.001
	Walk	20.65	0.438	0.411 to 0.464	<0.001
	All	20.92	0.447	0.432 to 0.463	<0.001
Berlei	**Rest**	**4.91**	**0.930**	**0.923 to 0.937**	**<0.001**
	**Warm Up**	**4.04**	**0.969**	**0.967 to 0.971**	**<0.001**
	**Run**	**2.28**	**0.972**	**0.970 to 0.973**	**<0.001**
	**Walk**	**3.59**	**0.946**	**0.943 to 0.948**	**<0.001**
	**All**	**3.31**	**0.978**	**0.978 to 0.979**	**<0.001**
Sensoria	**Rest**	**5.50**	**0.921**	**0.912 to 0.929**	**<0.001**
	Warm Up	9.98	0.582	0.556 to 0.608	<0.001
	Run	12.65	0.465	0.439 to 0.491	<0.001
	Walk	9.60	0.424	0.396 to 0.452	<0.001
	All	10.48	0.625	0.614 to 0.636	<0.001

Note: bold indicates having met the minimum established thresholds for each of the reliability tests.

**Figure 2:**
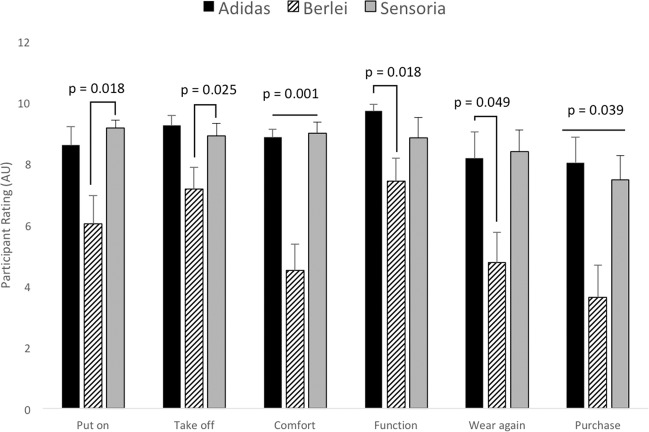
Subjective ratings (presented in arbitrary units, AU) of the Adidas Smart sports bra, the Berlei sports bra, and the Sensoria Fitness biometric sports bra. Participants provided perception on a scale of zero to ten, with zero being the lowest possible score, and ten being the greatest possible score. Data are presented as mean ± standard error, and differences between smart bras are noted by the p-value on the figure.

Subjective ratings of each smart bra are displayed in Fig. 2 (shown as mean ± standard error). Participants perceived the ease of putting on and taking off the Adidas and Sensoria smart bras to be equal. Participants felt the Berlei sports bra was more difficult to put on and take off compared to the Sensoria Fitness biometric sports bra. When comfort was considered, the Adidas and Sensoria smart bras were rated equally, and both were perceived to have greater comfort compared to the Berlei sport bra. The Adidas and Sensoria smart bras were rated to perform similarly with respect to bra function, and the Adidas smart bra was perceived by participants to have greater functionality compared to the Berlei sports bra. Participants responded that they were equally likely to wear both the Adidas and Sensoria smart bras, and were more likely to wear the Adidas smart bra compared to the Berlei sports bra. Lastly, when participants were asked whether they would purchase the smart bras for personal use, both the Adidas and Sensoria smart bras rated equally, and both were rated greater compared to the Berlei sport bra.

## Discussion

The purpose of the current investigation was to determine the validity and reliability of three different brands of heart rate sensing sports bras. It was hypothesized that each type of sports bra would be valid compared to a criterion measure and display consistency when compared to a second bout of the same exercise. The main finding is that the Berlei smart sports bra returned both valid and reliable heart rate measures across all conditions tested. However, participants also rated it the lowest on all perceptual measures of comfort and ease of use.

Validity of heart rate during activity has been described in wearable technology devices including smart shirts^3–7^, earbuds^8–10^ and sensors located on the forearm^11–13^ or the wrist^14–18^. This is the first investigation to report heart rate validity in sports bras during exercise. Perhaps the garments that most closely resemble the smart sports bras in the current study are heart rate sensing smart shirts. While smart shirts have been reported to be valid during laboratory-based walking (ICC range = 0.81 to 0.99)^4,7^ and cycling (ICC range = 0.98 to 0.99)^5^, they are much less accurate (ICC range = −0.012 to 0.354) when utilized in an outdoor environment during trail running exercise that involves more bodily movement^6^. Increased bodily movement, particularly at the breast, may explain the results of the current study. We found that our interpretation of validity for each model of sports bra depended on the manufacturer and the exercise condition. All heart rate sensing sports bras were considered valid during standing rest, which involves minimal movement. The Berlei and Sensoria sports bras were deemed to be valid during the walking condition, which generates relatively greater movement. However, only the Berlei sports bra was found to be valid across all exercise and movement conditions. It is possible that the design and composition of the Berlei sports bra, composed of greater amounts of polyester and elastine, was responsible for these favorable results.

While we have detailed the abundant heart rate validity investigations utilizing wearable technology, there is a relative dearth of studies reporting reliability even though such a need has been pointed out in the literature^2,37^. Investigations reporting heart rate reliability have evaluated smart shirts, which we believe to be the most similar type of wearable to the smart bras employed in the current study. In an investigation utilizing elite male cyclists, an ICC reliability range of 0.94 to 0.96 was reported during an incremental maximal test on a cycle ergometer at 50% and 75% of the maximal workload, respectively^5^. A different study utilizing both men and women walking on a treadmill at speeds between 40.2 m^.^min^−1^ and 80.5 m^.^min^−1^ reported Cronbach alpha reliability between 0.85 to 0.88^4^. Finally, an investigation using both men and women during self-paced trail hiking reported ICC reliability of 0.73 for heart rate, and 0.65 for maximal heart rate over a one mile distance^3^. It is tempting to speculate from these limited studies that reliability in heart rate measures derived from wearables is also influenced by amount of movement during exercise: stable cycle exercise returned the highest reliability measures, treadmill walking relatively lower, and walking on variable terrain the lowest consistency measures. In the current study, as movement demands increased, the reliability of the Sensoria Fitness biometric bra appeared to be impacted. The Sensoria sports bra was found to be reliable only during the resting condition. However, the Berlei sports bra returned consistent measures across all conditions.

While not the primary focus of this investigation, perception of each smart bra was collected from participants. A number of factors have been identified in the literature that affect the perception of sports bra comfort during exercise including breast asymmetry^38^, quality of support^39^, and design aspects accounting for cushioning^40^, strap placement^41^, and fabric and thermal properties^42,43^. Women who ran on a treadmill while wearing a high support sports bra rated their experience as more comfortable with less pain than while wearing a low support bra^39^. The bra strap contributes to the most overall discomfort during exercise^41^, and a cross-strap design with cushioning may help to reduce the distress^40^. As technology to measure biometric data such as heart rate is introduced to the sports bra garment, manufacturers should carefully consider design elements. It is interesting to note that in the current investigation the sports bra that was valid and reliable for heart rate in every condition, the Berlie sports bra, was also the lowest rated with respect to perceptual measures of comfort and ease of use.

This study is not without limitations. Previous studies have tested female participants at specific points in the menstrual cycle when the breast is in its lowest and most stable size^39^, and this was not taken into account in the current investigation. Additionally, a trained bra fitter was not employed^38^. While it is unlikely these considerations would have had an impact on the heart rate validity and reliability measures reported, the perceptual responses could have been impacted. Future studies should incorporate these factors into the study design, as well as whether look or appearance affected perception. In obtaining commercially available heart rate sensing sports bras, we did not control for the type of sensor each garment employed. The fabric sensor employed by the Adidas Smart sports bra returned valid measures at rest, but not during any of the exercise bouts. The Berlei sports bra and the Sensoria biometric sports bra both employed dual plastic flexible plastic sensors of the same size, geometry, and location on the underband of the garment. As these sports bras returned differing accuracy and consistency measures while utilizing the same conductive electrodes, it is unlikely that the sensors employed were responsible for the observed differences. Another potential limitation is that participants were allowed to self-select both their walking and running paces. It is felt that this limitation is somewhat mitigated, as self-selected speed positively affects both gait pattern and subsequently breast motion^40^. As the objective of the current investigation was to determine concurrent heart rate validity, we did not obtain or control for various physiological measures including sweat rate, metabolism, or skin temperature changes during the exercise bouts. A final limitation is that breast motion was not analyzed in the current investigation, which limits our ability to identify why some bras return more accurate and consistent heart rate measures across the various conditions. Future investigations of this nature would be needed to determine the impact of breast motion on heart rate validity and reliability in smart bras.

This is the first investigation to report heart rate validity and reliability in female participants who completed trials of self-paced walking and running while wearing smart sensing sports bras. The Adidas Smart sports bra was found to be valid during the rest condition but not reliable from one test to the next, the Berlei sports bra was both valid and reliable across all conditions, and the Sensoria Fitness biometric sports bra was valid and reliable at rest, and valid but not reliable during walking. Overall, participant perception of the smart sports bras was higher for items including comfort and function for the Adidas Smart sports bra and Sensoria Fitness sports bra, and lower for the Berlei sports bra. These findings highlight the need for sports bra manufacturers desiring to enter the wearable technology space for garments with both the ability to return accurate and reliable biometric data while at the same time providing the necessary function and comfort to females participating in physical activity.

## Methods

### Participants

Twenty-four healthy (not presenting with cardiovascular, metabolic, or renal disease; and no signs or symptoms suggestive of cardiovascular, metabolic, or renal disease)^44^ females volunteered to participate in the study. Recent heart rate validity literature utilizing smart garments have generally returned large effect sizes (0.81 to 0.99)^4,5,7^, however, to be conservative a moderate effect size (0.5) was utilized to determine that a total sample size of 21 would be sufficient. Participant descriptive characteristics included the following (mean ± SD): age = 22.2 ± 5.8 years, height = 174.6 ± 9.9 cm, mass = 71.2 ± 14.4 kg. Participants completed an informed consent document that was approved by the University of Nevada, Las Vegas Biomedical Sciences Institutional Review Board (protocol #997665), and methods were carried out in accordance with the relevant guidelines and regulations.

### Protocol

Participants completed 14-minute bouts of treadmill exercise (TMX428, Trackmaster, Newton, KS) which consisted of the following: standing on the treadmill for 1 minute, a 3-minute self-paced walking warm up, a self-paced 5-minute run, and a self-paced 5-minute walk. Participants determined pace as the treadmill speed they felt could be comfortably maintained for the duration of the 5-min bout. Each bout was completed twice while wearing the same sports bra in order to obtain reliability measures, and speed across trials was set the same as in the initial bout. Participants rested in a seated position between bouts until heart rate was within 10 bpm of their initial resting value, and then performed the second bout as previously described. As three different sports bras were utilized in the current study, participants performed a total of six exercise bouts in a randomized counterbalanced manner for garment. The garments utilized were:

Adidas Smart sports bra (Adidas Group, Herzogenaurach, Germany) – compression style sports bra; material composition = 86% nylon, 14% spandex; fabric heart rate sensors (2 ×9 cm) sewn into the underside of the underband on both the right and left sides of the garment; racerback with nonadjustable straps.

Berlei sports bra (Berlei Limited, Nottingham, England) – encapsulation/compression style sports bra; material composition = 47% polyamide, 37% polyester, 16% elastine; flexible plastic sensors (2 ×7.3 cm) located on the underside of the underband on both the right and left sides of the garment; racerback with adjustable straps.

Sensoria Fitness biometric sports bra (Sensoria Inc., Redmond, WA) – encapsulation/compression style sports bra; material composition = 74% polyamide, 18% polyester, 8% elastine; flexible plastic sensors (2 ×7.3 cm) located on the underside of the underband on both the right and left sides of the garment; racerback with nonadjustable straps.

The criterion measure was heart rate obtained using the Polar H7 heart rate monitor (Polar Electro, Kempele, Finland). Material composition = 38% polyamide, 29% polyurethane, 20% elastane, 13% polyester; single flexible plastic sensor (2.4 ×27.9 cm) worn concurrently and placed on the sternum just below the sports bra. The Polar H7 strap heart rate monitor has been utilized as a criterion measure for heart rate in several investigations^9,14,17,45^.

**Figure 3:**
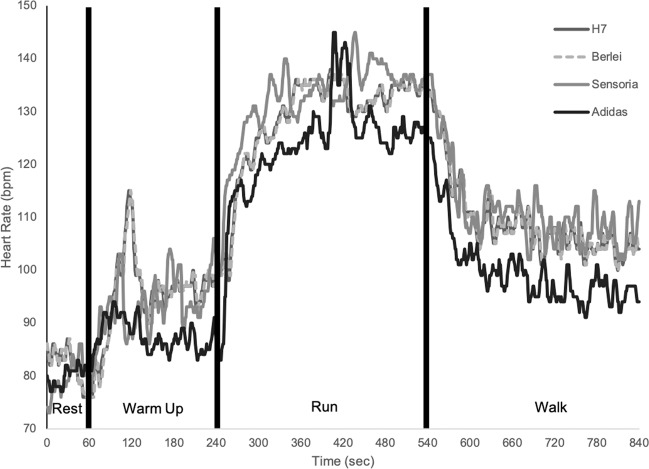
Representative example from a single participant, displaying heart rate responses obtained from the Polar H7 monitor (criterion), Adidas Smart sports bra, Berlei sports bra, and the Sensoria Fitness biometric sports bra.

All heart rate data were transmitted real time via Bluetooth to two synced iPad mini tablets (Apple Inc., Cupertino, CA) (one iPad mini was dedicated to the control Polar H7 strap, and the other iPad mini was dedicated to the Polar H7 receiver clipped into the respective smart bras) and captured into the Polar Flow application (Polar Electro, Kempele, Finland). The Polar H7 heart rate receiver has a sample rate of 1000 Hertz. Polar Flow reports heart rate as an average of the sampling rate in a second-by-second fashion, and can be exported via a comma-separated value spreadsheet file. This allowed for second-by-second synchronous evaluation between the criterion measure and the smart bras. The first exercise bout in which each smart bra was worn was utilized for validation purposes. Thus, 840 individual data points were obtained for each exercise bout, and 20160 data points across all participants for each garment. A representative tracing is shown in Fig. 3.

Perceptual measures were obtained immediately after the second bout (repeatability trial) after the participant took off the garment. The questionnaire asked participants to provide their subjective evaluation on a scale of zero to ten, with zero being the lowest possible score, and ten being the greatest possible score. Questions included “Ease of putting on the garment”, “Ease of taking off the garment”, “Overall comfort of the garment”, “Was the garment true to size”, “Did the garment function appropriately (i.e. secure, held in place)”, “Amount of chafing or other unpleasant sensations due to the garment”, “Would you wear this garment again”, and “Would you purchase this garment for personal use during exercise”.

### Statistical analysis

Validity was determined through three methods^2^: Mean Absolute Percent Error (MAPE), Bland-Altman bias and Limits of Agreement (LOA) for repeated samples with accompanying and 95% confidence intervals (CI), and Intraclass Correlations (ICC) (IBM SPSS, IBM Statistics version 24.0, Armonk, NY). MAPE considered acceptable if within 5%, and ICC acceptable if greater than 0.70 with a p-value was less than 0.05^46^.

Absolute reliability was determined through the Coefficient of Variation (CV), and considered consistent if less than 10%^46^. Test-retest reliability was determined through ICC analysis, with average measures ICC and 95% CI reported. Measures were considered to have reliability with ICC > 0.70 and p < 0.05.

To determine participant perceptual differences between garments, a one-way repeated measures ANOVA was conducted for each questionnaire item. All data were determined to satisfy the tests of homogeneity and sphericity. Differences between garments were determined *post hoc* using Bonferroni, and significance was accepted at p < 0.05.

## Availability of materials and data

This manuscript adheres to availability guidelines set forth by *Scientific Reports*.
